# The genome sequence of a snail-killing fly, *Coremacera marginata* (Fabricius, 1775)

**DOI:** 10.12688/wellcomeopenres.17482.1

**Published:** 2021-12-13

**Authors:** Olga Sivell, Duncan Sivell, Natural History Museum Genome Acquisition Lab, Darwin Tree of Life Barcoding collective, Wellcome Sanger Institute Tree of Life programme, Wellcome Sanger Institute Scientific Operations: DNA Pipelines collective, Tree of Life Core Informatics collective, Darwin Tree of Life Consortium

**Affiliations:** 1 Department of Life Sciences, Natural History Museum, London, UK

**Keywords:** Coremacera marginata, genome sequence, chromosomal, Diptera

## Abstract

We present a genome assembly from an individual female *Coremacera marginata *(Arthropoda; Insecta; Diptera; Sciomyzidae). The genome sequence is 980 megabases in span. The majority of the assembly (99.84%) is scaffolded into six chromosomal pseudomolecules, with the X sex chromosome assembled.

## Species taxonomy

Eukaryota; Metazoa; Ecdysozoa; Arthropoda; Hexapoda; Insecta; Pterygota; Neoptera; Endopterygota; Diptera; Brachycera; Muscomorpha; Sciomyzoidea; Sciomyzidae; Coremacera; *Coremacera marginata* (Fabricius, 1775) (NCBI:txid1226616).

## Background

Sciomyzidae (Diptera) are commonly known as snail-killing flies or marsh flies, the latter name reflecting the habitat preference of many species from this family. *Coremacera marginata* (Diptera, Sciomyzidae) is a dark grey-brown fly with a characteristic wing pattern consisting of a strongly infuscated wing margin (darker near the coastal vein) and dark brown base colour with numerous pale spots across the rest of the wing. The species is fairly common and widely distributed in England and Wales, in Scotland it has only been recorded from around the Moray and Dornoch Firths ( Ball, 2017). It prefers open and dry habitats, particularly calcareous grasslands, also coastal dunes, open scrubby woods, old fields on woodland margins and is occasionally found in wetland habitats ( Ball, 2017; Rozkošný, 1984). Flight period occurs from mid-May till beginning of October ( Ball, 2017; Speight & Knutson, 2012).

*Coremacera marginata* is oviparous. The eggs are laid on or near the host. The larvae are parasitoids of terrestrial snails ( Knutson, 1970; Rozkošný, 1984; Rozkošný, 1987), with a preference of *Cochlicopa* and *Discus* species in laboratory conditions ( Knutson, 1973; Rozkošný, 1984). Upon hatching the larva feeds on a living snail. The host survives for up to ten days, unless infested with multiple larvae (up to 11 have been reported to attack a single snail), in which case death can occur within 24 hours ( Knutson, 1973; Rozkošný, 1984). The larva continues to feed on the decomposing tissues until it reaches the second or third instar. It then moves to a second snail to continue feeding, killing the host in one to two days. Rarely, the larva will require a third snail to complete its development. Pupation occurs outside the shell. The larval stage lasts from 22 to 97 days with an average of 52 days, and the pupa from 47 to 124 days ( Knutson, 1973; Rozkošný, 1984). This species overwinters as a mature larva or as a pupa ( Ball, 2017; Speight & Knutson, 2012). Adults feed on flowers, dead insects and snails, and also on insect eggs and live snails’ secretions ( Berg & Knutson, 1978). First and third larval instars and the puparium have been described by Knutson (1973).

*Coremacera marginata* was split into two subspecies, *Coremacera marginata marginata* (Fabricius, 1775) and *Coremacera marginata pontica*, by Elberg (1968) based on paler specimens from southern European Russia and Iran. This was subsequently rejected by Knutson (1973) due to a lack of differentiating structural characters that would support the separation.

The high-quality genome sequence described here is the first one reported for *Coremacera marginata* and has been generated as part of the Darwin Tree of Life project. It will aid in understanding the biology, physiology and ecology of the species.

## Genome sequence report

**Figure 1.**
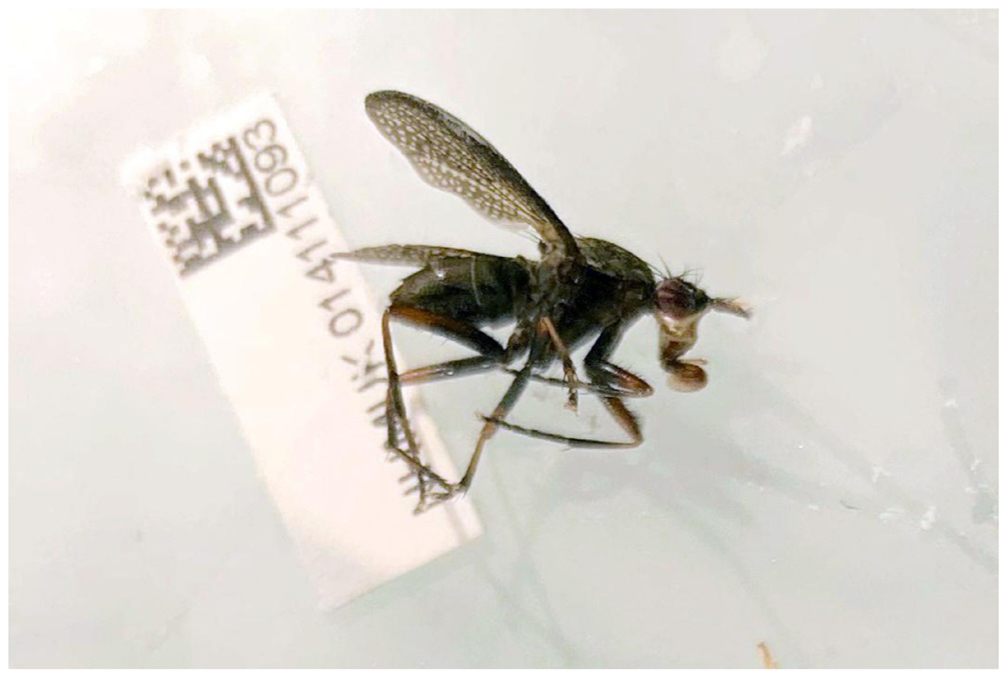
Image of the idCorMarg1 specimen taken during preservation and processing.

The genome was sequenced from a single female *C. marginata* ( Figure 1) collected from Wigmore Park, Luton, UK (latitude 51.88378, longitude -0.36861422). A total of 25-fold coverage in Pacific Biosciences single-molecule long reads and 33-fold coverage in 10X Genomics read clouds were generated. Primary assembly contigs were scaffolded with chromosome conformation Hi-C data. Manual assembly curation corrected 617 missing/misjoins and removed 8 haplotypic duplications, reducing the assembly size by 0.18% and the scaffold number by 82.91%, and increasing the scaffold N50 by 268.04%.

**Figure 2.**
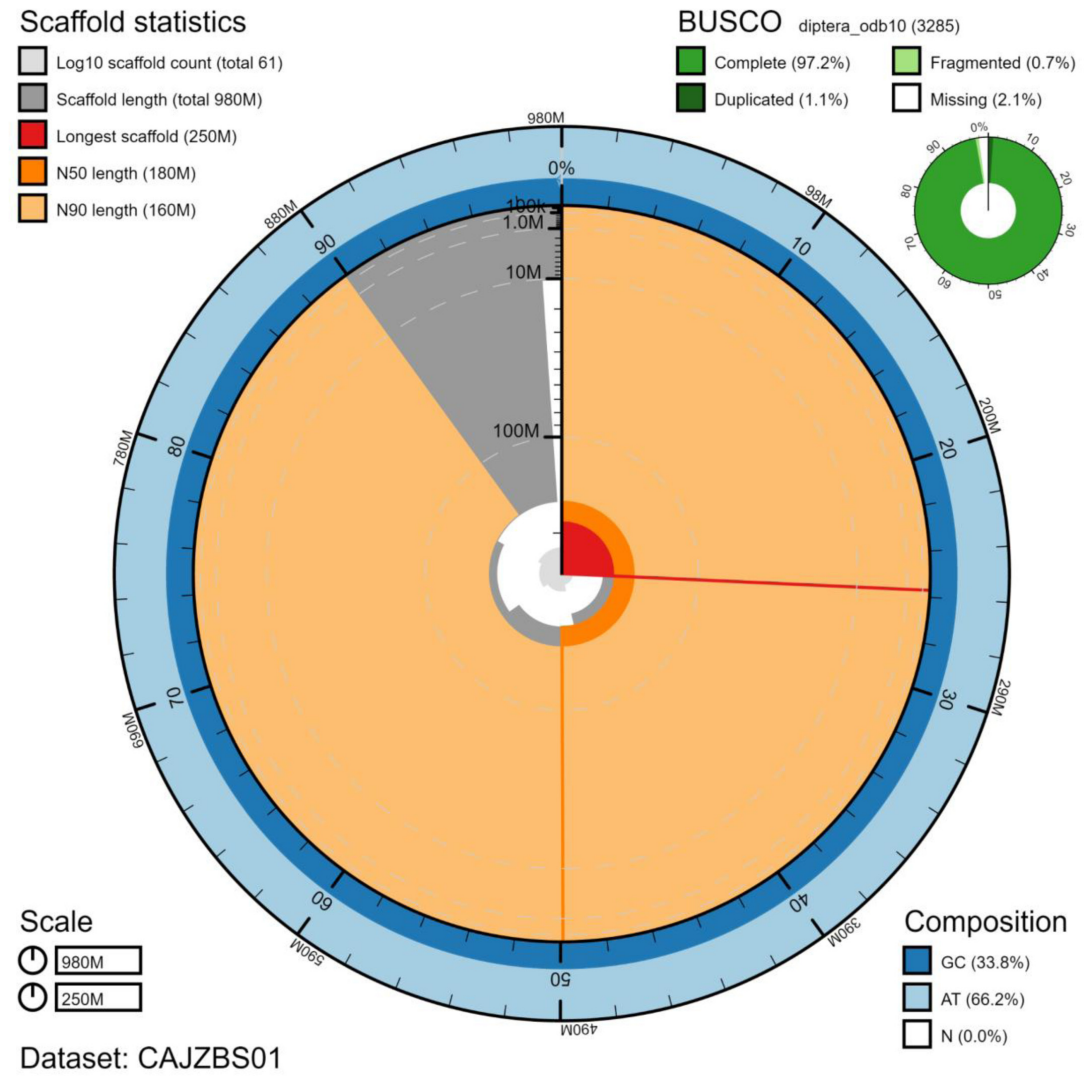
Genome assembly of *Coremacera marginata*, idCorMarg1.1: metrics. The BlobToolKit Snailplot shows N50 metrics and BUSCO gene completeness. The main plot is divided into 1,000 size-ordered bins around the circumference with each bin representing 0.1% of the 979,680,483 bp assembly. The distribution of chromosome lengths is shown in dark grey with the plot radius scaled to the longest chromosome present in the assembly (252,586,203 bp, shown in red). Orange and pale-orange arcs show the N50 and N90 chromosome lengths (184,110,224 and 162,576,309 bp), respectively. The pale grey spiral shows the cumulative chromosome count on a log scale with white scale lines showing successive orders of magnitude. The blue and pale-blue area around the outside of the plot shows the distribution of GC, AT and N percentages in the same bins as the inner plot. A summary of complete, fragmented, duplicated and missing BUSCO genes in the diptera_odb10 set is shown in the top right. An interactive version of this figure is available at https://blobtoolkit.genomehubs.org/view/idCorMarg1.1/dataset/CAJZBS01/snail.

The final assembly has a total length of 980 Mb in 60 sequence scaffolds with a scaffold N50 of 184.1 Mb ( Table 1). The majority, 99.84%, of the assembly sequence was assigned to 6 chromosomal-level scaffolds, representing 5 autosomes (numbered by sequence length), and the X sex chromosome ( Figure 2– Figure 5; Table 2). The assembly has a BUSCO v5.1.2 ( Manni *et al.*, 2021) completeness of 97.2% (single 96.2%, duplicated 1.1%) using the diptera_odb10 reference set. While not fully phased, the assembly deposited is of one haplotype. Contigs corresponding to the second haplotype have also been deposited.

**Table 1.** Genome data for *Coremacera marginata*, idCorMarg1.1.

*Project accession data*	
Assembly identifier	idCorMarg1.1
Species	*Coremacera marginata*
Specimen	idCorMarg1
NCBI taxonomy ID	1226616
BioProject	PRJEB45188
BioSample ID	SAMEA7521524
Isolate information	Female, thorax (genome assembly), head (Hi-C), abdomen (RNA-Seq)
*Raw data accessions*	
PacificBiosciences SEQUEL II	ERR6412041
10X Genomics Illumina	ERR6054930-ERR6054933
Hi-C Illumina	ERR6054934
Illumina polyA RNA-Seq	ERR6688408
*Genome assembly*	
Assembly accession	GCA_914767935.1
*Accession of alternate haplotype*	GCA_914767655.1
Span (Mb)	980
Number of contigs	889
Contig N50 length (Mb)	2.7
Number of scaffolds	60
Scaffold N50 length (Mb)	184.1
Longest scaffold (Mb)	252.6
BUSCO * genome score	C:97.2%[S:96.2%,D:1.1%],F:0.7%,M:2.1%, n:3285

*BUSCO scores based on the diptera_odb10 BUSCO set using v5.1.2. C= complete [S= single copy, D=duplicated], F=fragmented, M=missing, n=number of orthologues in comparison. A full set of BUSCO scores is available at https://blobtoolkit.genomehubs.org/view/idCorMarg1.1/dataset/CAJZBS01/busco.

**Figure 3.**
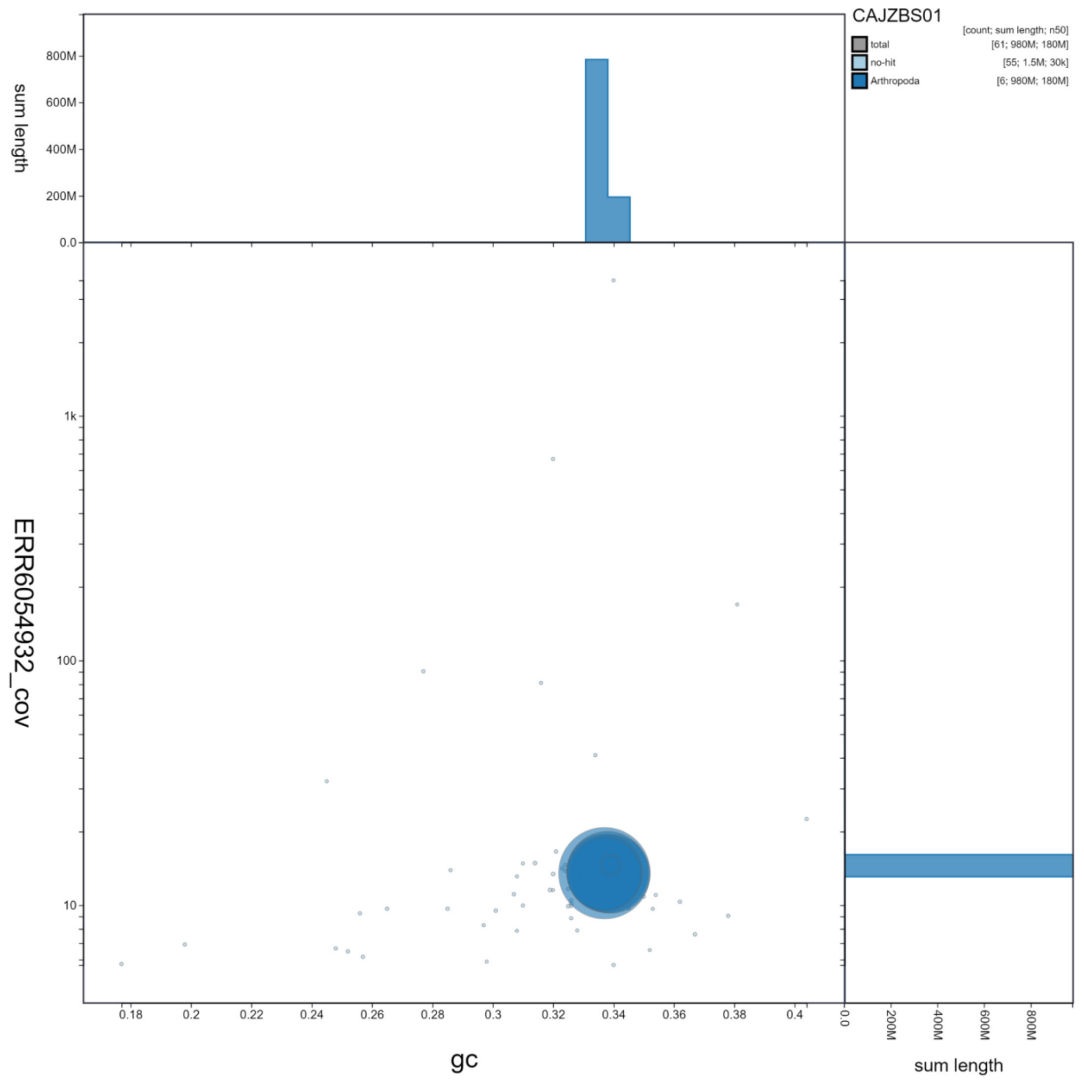
Genome assembly of *Coremacera marginata*, idCorMarg1.1: GC coverage. BlobToolKit GC-coverage plot. Scaffolds are coloured by phylum. Circles are sized in proportion to scaffold length. Histograms show the distribution of scaffold length sum along each axis. An interactive version of this figure is available at https://blobtoolkit.genomehubs.org/view/idCorMarg1.1/dataset/CAJZBS01/blob.

**Figure 4.**
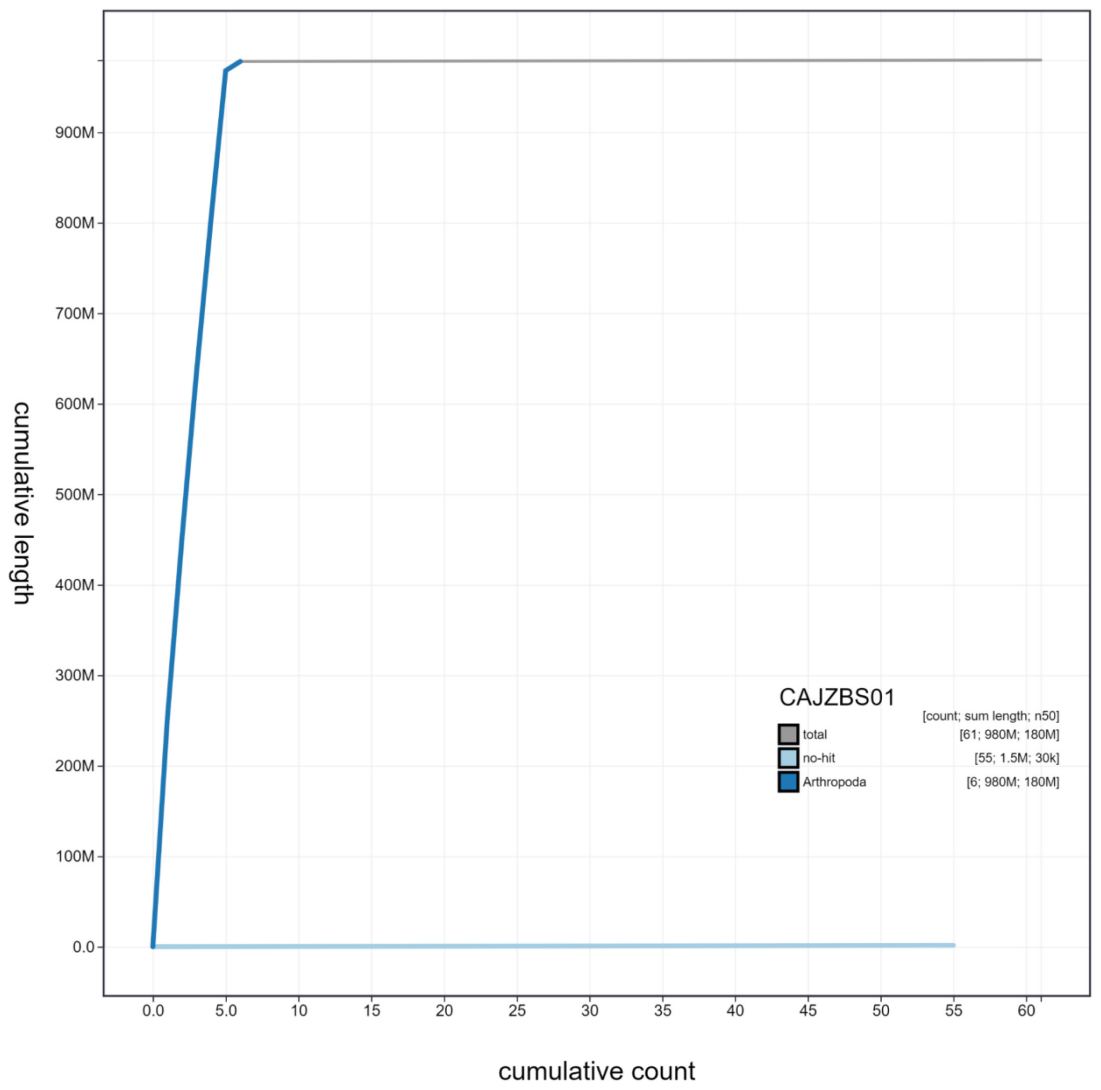
Genome assembly of *Coremacera marginata*, idCorMarg1.1: cumulative sequence. BlobToolKit cumulative sequence plot. The grey line shows cumulative length for all scaffolds. Coloured lines show cumulative lengths of scaffolds assigned to each phylum using the buscogenes taxrule. An interactive version of this figure is available at https://blobtoolkit.genomehubs.org/view/idCorMarg1.1/dataset/CAJZBS01/cumulative.

**Figure 5.**
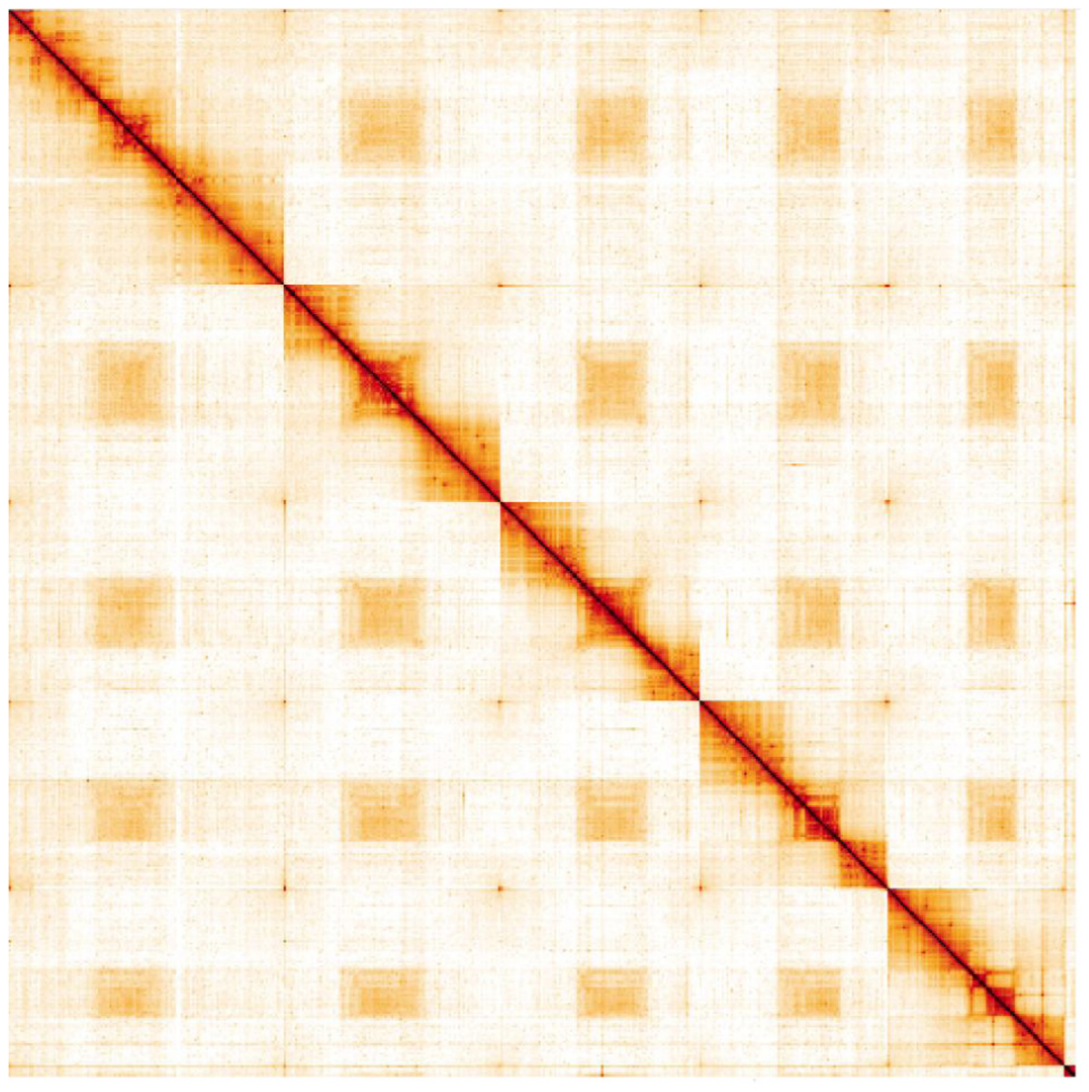
Genome assembly of *Coremacera marginata*, idCorMarg1.1: Hi-C contact map. Hi-C contact map of the idCorMarg1.1 assembly, visualised in HiGlass. Chromosomes are given in order of size, from left to right and top to bottom.

**Table 2.** Chromosomal pseudomolecules in the genome assembly of *Coremacera marginata*, idCorMarg1.1.

INSDC accession	Chromosome	Size (Mb)	GC%
OU612043.1	1	252.59	33.7
OU612044.1	2	198.14	33.8
OU612045.1	3	184.11	33.9
OU612046.1	4	170.64	33.7
OU612047.1	5	162.58	33.7
OU612048.1	X	10.12	33.9
OU612049.1	MT	0.02	34.6
-	Unplaced	1.49	31.4

## Methods

### Sample acquisition and nucleic acid extraction

A female *C. marginata* (idCorMarg1) was collected from Wigmore Park, Luton, UK (latitude 51.88378, longitude -0.36861422) by Olga Sivell, Natural History Museum, and identified by Duncan Sivell, Natural History Museum based on Rozkošný (1984) and Ball (2017). The specimens were collected using a net and snap-frozen on dry ice.

DNA was extracted at the Tree of Life laboratory, Wellcome Sanger Institute. The idCorMarg1 sample was weighed and dissected on dry ice with tissue set aside for Hi-C and RNA sequencing. Thorax tissue was cryogenically disrupted to a fine powder using a Covaris cryoPREP Automated Dry Pulveriser, receiving multiple impacts. Fragment size analysis of 0.01-0.5 ng of DNA was then performed using an Agilent FemtoPulse. High molecular weight (HMW) DNA was extracted using the Qiagen MagAttract HMW DNA extraction kit. Low molecular weight DNA was removed from a 200-ng aliquot of extracted DNA using 0.8X AMpure XP purification kit prior to 10X Chromium sequencing; a minimum of 50 ng DNA was submitted for 10X sequencing. HMW DNA was sheared into an average fragment size between 12–20 kb in a Megaruptor 3 system with speed setting 30. Sheared DNA was purified by solid-phase reversible immobilisation using AMPure PB beads with a 1.8X ratio of beads to sample to remove the shorter fragments and concentrate the DNA sample. The concentration of the sheared and purified DNA was assessed using a Nanodrop spectrophotometer and Qubit Fluorometer and Qubit dsDNA High Sensitivity Assay kit. Fragment size distribution was evaluated by running the sample on the FemtoPulse system.

RNA was extracted from abdomen tissue in the Tree of Life Laboratory at the WSI using TRIzol (Invitrogen), according to the manufacturer’s instructions. RNA was then eluted in 50 μl RNAse-free water and its concentration RNA assessed using a Nanodrop spectrophotometer and Qubit Fluorometer using the Qubit RNA Broad-Range (BR) Assay kit. Analysis of the integrity of the RNA was done using Agilent RNA 6000 Pico Kit and Eukaryotic Total RNA assay.

### Sequencing

Pacific Biosciences HiFi circular consensus and 10X Genomics read cloud DNA sequencing libraries were constructed according to the manufacturers’ instructions. Poly(A) RNA-Seq libraries were constructed using the NEB Ultra II RNA Library Prep kit. DNA and RNA sequencing was performed by the Scientific Operations core at the WSI on Pacific Biosciences SEQUEL II (HiFi), Illumina HiSeq X (10X) and Illumina HiSeq 4000 (RNA-Seq) instruments. Hi-C data were generated from abdomen tissue of the same specimen using the Arima Hi-C+ kit and sequenced on an Illumina NovaSeq 6000 instrument.

### Genome assembly

Assembly was carried out with Hifiasm ( Cheng *et al*., 2021); haplotypic duplication was identified and removed with purge_dups ( Guan *et al*., 2020). One round of polishing was performed by aligning 10X Genomics read data to the assembly with longranger align, calling variants with freebayes ( Garrison & Marth, 2012). The assembly was then scaffolded with Hi-C data ( Rao *et al*., 2014) using SALSA2 ( Ghurye *et al*., 2019). The assembly was checked for contamination as described previously ( Howe *et al*., 2021). Manual curation ( Howe *et al*., 2021) was performed using HiGlass ( Kerpedjiev *et al*., 2018) and Pretext. The mitochondrial genome was assembled using MitoHiFi ( Uliano-Silva *et al*., 2021), which performed annotation using MitoFinder ( Allio *et al*., 2020). The genome was analysed and BUSCO scores generated within the BlobToolKit environment ( Challis *et al*., 2020). Table 3 contains a list of all software tool versions used, where appropriate.

**Table 3.** Software tools used.

Software tool	Version	Source
Hifiasm	0.12	Cheng *et al.,* 2021
purge_ dups	1.2.3	Guan *et al.,* 2020
SALSA2	2.2	Ghurye *et al.,* 2019
longranger align	2.2.2	https:// support.10xgenomics.com/ genome-exome/software/ pipelines/latest/advanced/ other-pipelines
freebayes	1.3.1-17-gaa2ace8	Garrison & Marth, 2012
MitoHiFi	2.0	Uliano-Silva *et al.,* 2021
gEVAL	N/A	Chow *et al.,* 2016
HiGlass	1.11.6	Kerpedjiev *et al.,* 2018
PretextView	0.2.x	https://github.com/wtsi- hpag/PretextView
BlobToolKit	2.6.2	Challis *et al.,* 2020

### Ethics/compliance issues

The materials that have contributed to this genome note have been supplied by a Darwin Tree of Life Partner. The submission of materials by a Darwin Tree of Life Partner is subject to the Darwin Tree of Life Project Sampling Code of Practice. By agreeing with and signing up to the Sampling Code of Practice, the Darwin Tree of Life Partner agrees they will meet the legal and ethical requirements and standards set out within this document in respect of all samples acquired for, and supplied to, the Darwin Tree of Life Project. Each transfer of samples is further undertaken according to a Research Collaboration Agreement or Material Transfer Agreement entered into by the Darwin Tree of Life Partner, Genome Research Limited (operating as the Wellcome Sanger Institute), and in some circumstances other Darwin Tree of Life collaborators.

## Data availability

European Nucleotide Archive: Coremacera marginata. Accession number PRJEB45188: https://www.ebi.ac.uk/ena/browser/view/PRJEB45188.

The genome sequence is released openly for reuse. The *C. marginata* genome sequencing initiative is part of the Darwin Tree of Life (DToL) project. All raw sequence data and the assembly have been deposited in INSDC databases. The genome will be annotated using the RNA-Seq data and presented through the Ensembl pipeline at the European Bioinformatics Institute. Raw data and assembly accession identifiers are reported in Table 1.

## Author information

Members of the Natural History Museum Genome Acquisition Lab are listed here: https://doi.org/10.5281/zenodo.5746819.

Members of the Darwin Tree of Life Barcoding collective are listed here: https://doi.org/10.5281/zenodo.5744972.

Members of the Wellcome Sanger Institute Tree of Life programme are listed here: https://doi.org/10.5281/zenodo.5744840.

Members of Wellcome Sanger Institute Scientific Operations: DNA Pipelines collective are listed here: https://doi.org/10.5281/zenodo.5746904.

Members of the Tree of Life Core Informatics collective are listed here: https://doi.org/10.5281/zenodo.5743293.

Members of the Darwin Tree of Life Consortium are listed here: https://doi.org/10.5281/zenodo.5638618.
