# Supplementary material for: The genome sequence of a snail-killing fly,  Coremacera marginata (Fabricius, 1775)
Source: Wellcome Open Res. 2021 Dec 13;6:338. [Version 1] doi: 10.12688/wellcomeopenres.17482.1 (PMC9647149; doi:10.12688/wellcomeopenres.17482.1)
Supplement: Peer review history

# Peer review: Reviewer response for version 1

**DOI:** 10.21956/wellcomeopenres.19332.r53078

**Published:** 2022-11-09

**Authors:** Craig Wilding

**Affiliations:** 1 School of Biological and Environmental Sciences, Liverpool John Moores University, Liverpool, UK

This Genome Note provides a useful review of the genome assembly and annotation efforts for *Coremacera marginata*. Sivell and Sivell have produced an exceptionally high-quality genome for a single female fly. The methodological details are clear and the results well presented. Personally, I think It would be useful to know (in the introduction) what pre-existing genomic or transcriptomic resources exist for the Sciomyzids? Is this the first genome available? Indeed is the genome size typical or exceptional for this group? I assume that as for most other flies the male is heterogametic so it is unfortunate that a male was not sequenced. Perhaps a comment to this effect could be added to the discussion.

 RNASeq data are mentioned but there is no indication of the number of gene models identified. Is RNASeq data the only source of gene models or was automatic annotation also used? Perhaps the number of gene models could be added to Table 1?

 I would also find it interesting to have further details of the manual curation that led to the improvement in scaffold number and scaffold N50.

Are sufficient details of methods and materials provided to allow replication by others?

Yes

Is the rationale for creating the dataset(s) clearly described?

Yes

Are the datasets clearly presented in a useable and accessible format?

Yes

Are the protocols appropriate and is the work technically sound?

Yes

Reviewer Expertise:

Evolutionary genetics and genomics (invertebrates)

I confirm that I have read this submission and believe that I have an appropriate level of expertise to confirm that it is of an acceptable scientific standard.

# Peer review: Reviewer response for version 1

**DOI:** 10.21956/wellcomeopenres.19332.r49256

**Published:** 2022-04-07

**Authors:** Liping Yan

**Affiliations:** 1 School of Ecology and Nature Conservation, Beijing Forestry University, Beijing, China

Sivell et al. (2021) sequenced genomic data of the snail-killing fly, Coremacera marginata. The study was well planned, the methodology is fine, and manuscript is well structured and written in general. Although this is a small paper documenting data, I am looking forward to the follow-up studies with further information and the evolutionary story of this interesting fly and its relatives.

Are sufficient details of methods and materials provided to allow replication by others?

Yes

Is the rationale for creating the dataset(s) clearly described?

Yes

Are the datasets clearly presented in a useable and accessible format?

Yes

Are the protocols appropriate and is the work technically sound?

Yes

Reviewer Expertise:

Phylogenomics, transcriptomics.

I confirm that I have read this submission and believe that I have an appropriate level of expertise to confirm that it is of an acceptable scientific standard.
